# Mortality of patients with sepsis in intensive care units at tertiary hospitals in Jordan: Prospective cohort study

**DOI:** 10.1097/MD.0000000000040169

**Published:** 2024-10-25

**Authors:** Saleh Al Omar, Jafar Alasad Alshraideh, Islam Oweidat, Mohammad Al Qadire, Atika Khalaf, Yasmeen Abu Sumaqa, Khalid Al-Mugheed, Amany Anwar Saeed Alabdullah, Sally Mohammed Farghaly Abdelaliem

**Affiliations:** a Faculty of Nursing, Al-Balqa Applied University, Salt, Jordan; b Acute and Critical Care, University of Jordan, Amman, Jordan; c Community and Mental Health Nursing Department, Faculty of Nursing, Zarqa University, Zarqa, Jordan; d College of Nursing, Sultan Qaboos University, Muscat, Oman; e Faculty of Nursing, Al Al-Bayt University, Mafraq, Jordan; f The PRO-CARE Group, Faculty of Health Science, Kristianstad University, Kristianstad, Sweden; g Department of Nursing, Fatima College of Heath Sciences, Ajman, United Arab Emirates; h Faculty of Nursing, Al-Balqa Applied University, Salt, Jordan; i College of Nursing, Riyadh Elm University, Riyadh, Saudi Arabia; j Department of Maternity and Pediatric Nursing College of Nursing, Princess Nourah bint Abdulrahman University, Riyadh, Saudi Arabia; k Department of Nursing Management and Education, College of Nursing, Princess Nourah bint Abdulrahman University, Riyadh, Saudi Arabia.

**Keywords:** intensive care, mortality, predictors, sepsis, septic shock

## Abstract

The aim of this study was to describe the 30-day mortality rate of adult patients with sepsis and septic shock in 6 intensive care units of 2 tertiary hospitals in Jordan. A prospective cohort design was used. Patients with sepsis and septic shock admitted to the medical and surgical intensive care units at 2 tertiary hospitals were followed up during the period between February 2022 and June 2022 (N = 148). Data were analyzed using SPSS, version 23. Moreover, descriptive statistics, chi-square, and binary logistic regression were used. Notably, 52.7% of patients with sepsis and septic shock died within 30 days of diagnosis of sepsis and septic shock. Sequential Organ Failure Assessment score and the history of having solid tumors significantly predicted the 30-day mortality rate. Moreover, 43 (29.0%) patients with sepsis and septic shock had positive blood cultures, and 46 (31.0%) had positive urine cultures. Patients with sepsis and septic shock have a notable mortality rate that can be predicted from total Sequential Organ Failure Assessment scores and from the history of having solid tumors. Early assessment and initiation of treatment for sepsis essentially would reduce the likelihood of progression of sepsis to septic shock and would reduce associated patients’ mortality.

## 1. Introduction

Sepsis is an abnormal response of the immune system to infection; however, septic shock includes circulatory, metabolic, and cellular abnormalities that can increase the risk of mortality more than sepsis does alone.^[1]^ It is well-documented that sepsis can increase patients’ morbidity, length of stay, and mortality rate.^[2]^ Specifically, 41.9% of patients with sepsis died inside hospitals.^[3]^ Delay in recognition and treatment of sepsis was associated with increased hospital mortality rate and length of stay.^[4]^ One-third of the patients who died in hospitals in the United States of America were diagnosed with severe sepsis.^[5]^ According to different studies, the mortality rate for patients with sepsis ranged between 10.0% and 52.0%, and the high mortality rate was caused by septic shock.^[6]^ The rate of 30-day mortality among patients with sepsis was 40.0%, as reported in a prospective descriptive study conducted over 6 years in an intensive care unit (ICU) in Macedonia.^[7]^ The mortality rate was also 23.0% for patients with sepsis and 33% for patients with septic shock in an ICU of a tertiary hospital in Australia^[8]^ and 21.0% in a large study (N = 28,752) conducted in Thailand.^[9]^

Several factors were found to increase the 30-day mortality rate among patients with sepsis; this includes severe hyperglycemia, high glycemic variability, sepsis-associated encephalopathy,^[10]^ high plasma calprotectin level, lower platelet count, higher plasma autotoxin activity, increased lactate level, increased age, hospital-acquired sepsis, and remaining hypotensive after receiving vasopressors.^[11]^

Early detection of sepsis onset by physicians and nurses can improve outcomes and reduce mortality.^[12]^ The in-hospital sepsis-related mortality rate was reduced significantly over 7 years of applying a quality improvement initiative.^[13]^ In a recent study, the ICU mortality rate of adult patients with sepsis in a tertiary hospital in Jordan was 57.8%.^[14]^ But the 30-day mortality rate is a valuable indicator used in health care research.^[15,16]^ However, it was not assessed among adult patients with sepsis in Jordan; the current study sought to answer this gap of knowledge. As expected, this study will broaden the horizons of the awareness of nursing educators, physicians, administrators, researchers, and clinicians regarding this crucial issue in Jordan and globally. The current study is an attempt to assess the extent of the problem and report the outcome of patients with sepsis. It sought to answer this gap of knowledge. Therefore, the purpose of this study was to describe the 30-day mortality rate and associated factors among adult patients with sepsis and septic shock in 6 ICUs in 2 tertiary Jordanian hospitals.

## 2. Materials and methods

### 2.1. Study design

A prospective cohort design was used, in which 148 adult patients admitted to the ICUs were followed for 30 days, including the period of hospitalization.

### 2.2. Settings

The study was conducted in 2 tertiary hospitals; the first one is in the middle of the country and has a capacity of 582 beds, 49 of which are adult ICU beds. Around 716 patients are admitted into this hospital’s 4 adult ICUs yearly. The second hospital is in the northern area of Jordan with a general capacity of 651 beds, 28 of which are adult ICU beds. Both hospitals are referral public hospitals serving urban or rural populations. However, patients can also be admitted directly to the hospitals. These hospitals were selected for the following reasons: they are among the biggest hospitals in the country, they provide a wide range of health care services and have many specialties, the hospitals have the highest admission rate in the country, they are accredited by the Joint Commission International, and they are convenient to the researchers. Both hospitals use the Surviving Sepsis Campaign guidelines for the diagnosis and treatment of sepsis and septic shock. However, they do not have a systematic way to monitor compliance with the treatment guidelines. They monitor compliance with the diagnostic criteria only. There were different characteristics between the ICUs of the 2 hospitals (see Table 1).

**Table 1:** Different characteristics of the hospitals’ ICUs.

Characteristic	Hospital number one	Hospital number two
Total number of adult ICUs in the hospital	Seven	Seven
Total number of selected adult ICUs from which data were collected	Six	Two
Types of ICUs	Medical ICU Surgical ICUs Surgical ICU (postoperative, used for patients with major surgeries whose conditions deteriorate or need further monitoring) Mixed (a post-interventional ICU for patients with the most critical medical and surgical illnesses. It has a separate room for each patient)	Medical ICU Surgical ICU
Total number of beds in the selected ICUs	49*	28
Does all ICUs have isolation rooms?	Yes	Yes

ICU = intensive care unit.

* Not all beds were used for admitting patients due to maintenance issues.

### 2.3. Sample and sample size

A convenient sample was enrolled for this study. Patients were eligible to participate in the study if they had confirmed sepsis or septic shock and were admitted to the 6 medical and surgical ICUs of the 2 hospital settings between February 13, 2022, and May 13, 2022. In addition, they must be 18 years or older and agree to take part in the study. However, patients were excluded if they refused to participate in the study.

### 2.4. Instruments

**Figure 1.**
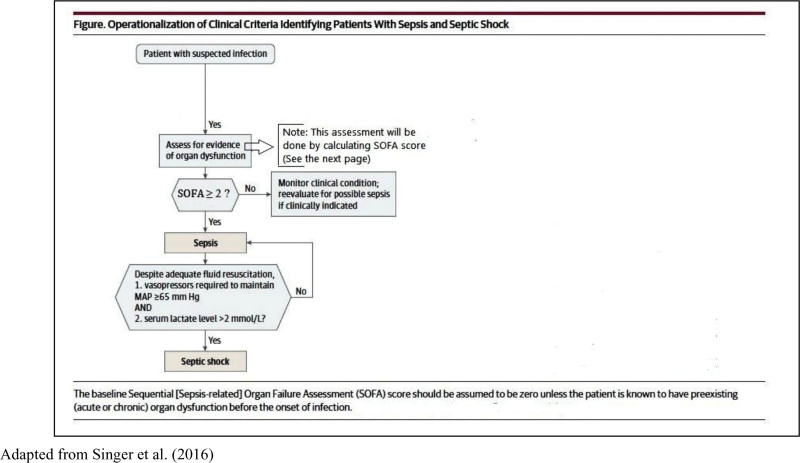
Sepsis/septic shock screening flowchart. Patient’s Medical Record Number: ______________Name and signature of the staff nurse: ______________________________Site of the suspected infection (Body organ): ____________________Date: ____/ ____/ 20____, Time: ___: ___ (24 hr clock) (date and time of sepsis or septic shock diagnosis). Note: Please circle the applicable words (Yes/No) on the flowchart.

Data were collected using 2 instruments. The first instrument was the Operationalization of Clinical Criteria Identifying Patients with Sepsis and Septic Shock. It is a flowchart adapted from the Sepsis-3 task force study in 2016 and used in the 2 hospitals^[1]^ (see Figure 1). This instrument consisted of 2 parts: the first one was to assess whether patients have sepsis or septic shock. The second part contained a numerical scale used to assess the degree of organ/system failure on a scale ranging from 0 to 4 for each body organ/system. Based on this instrument, patients with documented or suspected infection and a Sequential Organ Failure Assessment (SOFA) score equal to or greater than 2 were considered to have sepsis. Patients with sepsis who required vasopressor administration and whose serum lactate was > 2 mmol/L despite adequate fluid administration were considered to have septic shock. The total score can range between 0 and 24. The body organs/systems assessed using this instrument included the respiratory, coagulation, liver, cardiovascular, renal, and central nervous system.

The second instrument was the Charlson Comorbidity Index, which assesses the risk of patients’ mortality.^[17]^ Sixteen diseases were assessed using this instrument, with scores ranging from 0 to 6 for each item, and the total score could range from 0 to 34. Diseases assessed include myocardial infarction, congestive heart failure, peripheral vascular disease, cerebrovascular disease, dementia, chronic obstructive pulmonary disease, connective tissue disease, peptic ulcer disease, diabetes mellitus, moderate to severe chronic kidney disease, hemiplegia, leukemia, malignant lymphoma, solid tumor, liver disease, and acquired immunodeficiency syndrome. The Charlson Comorbidity Index has high (0.9) inter-rater reliability^[18]^ and high predictability of 30-day mortality of patients with septic shock.^[19]^ Further information about patients’ demographical and clinical characteristics was collected, which included age, sex, and microbiological culture results.

### 2.5. Data collection

Ethical approvals to conduct the study were obtained from the Institutional Review Boards at the University of Jordan and King Abdullah University Hospital Ethical Review Board (Ref. No. 90/2022/54 and Ref. No. 13/1/640). All participants/participants’ families gave written informed consent, and all methods were carried out in accordance with the guidelines and regulations of the Declaration of Helsinki. Data were collected between February 2022 and June 2022. Patients were assessed as soon as possible after being admitted within 24 hours of admission to the ICU. Moreover, patients were screened for sepsis and septic shock every day during the period of data collection. Patients who had more than 1 episode of sepsis during their admission were considered as 1 case. Because the study’s main aim was to assess the mortality rate, a patient with sepsis was considered included in the case without considering the recurrence of sepsis following the first episode. Furthermore, patients who were suspected to have sepsis but then had another diagnosis confirmed were excluded based on the diagnostic instrument.

The researchers and the attending ICU physicians collaborated on screening for sepsis and septic shock. The researchers reviewed the patients’ charts to obtain further clinical information and document contact details for follow-up. Also, patients’ mobile phone numbers were obtained to contact them after 30 days of sepsis diagnosis, seeking information about 30-day mortality. Furthermore, patients’ records were rechecked every week after discharge to confirm any other new or relevant information regarding mortality and diagnostic studies. Patients were followed during hospitalization and for 30 days from sepsis diagnosis if they were discharged alive from the hospital. The endpoint was 30-day mortality; the last patient was contacted in June 2022.

### 2.6. Data analysis

Data were analyzed using SPSS, version 23. Descriptive statistics were used to describe sample characteristics and clinical variables. A chi-square was used to test for differences in demographic and clinical variables between patients with sepsis who died and those who remained alive at 30 days. Binary logistic regression was performed to assess predictors of 30-day mortality among patients with sepsis. Variables were selected based on the literature review and the result of the binary correlation between all variables.

Two scores were missing for the “respiration” item of the SOFA scale; this happened because 2 partial pressure of oxygen results were not obtained at an early stage to be considered in the calculation of total SOFA scores. However, the total SOFA scores of these 2 patients were higher than 2, even without adding the partial pressure of oxygen results into the total SOFA score. Consequently, these 2 patients were considered eligible to participate in the study.

## 3. Results

A total number of 914 patients were admitted to adult medical, surgical, and mix ICUs of the selected hospitals over 3 months, divided into 489 (53.5%) patients in the hospital setting number 1 and 425 (46.49%) patients in the hospital setting number 2. All these 914 patients were examined for sepsis and septic shock diagnostic tools. The number of patients potentially eligible for sepsis and septic shock was 183. However, 31 of them were excluded because they did not have a confirmed diagnosis of sepsis or septic shock. In addition, 4 patients declined participation in the study. At the end of the 3 months, 148 adult patients were confirmed as eligible patients with sepsis. The response rate was 97%. These patients were followed up until the end of data collection, and their data were analyzed (see Figure 1).

The study participants consisted of 148 patients with sepsis septic shock. Seventy-four (50%) of patients had septic shock, and 74 (50%) had sepsis only. The mean age of participants was 66.1 years (SD = 17.5). Females constituted 81 (54.7%) of patients. Seventy-three (49.3%) patients were admitted to medical ICUs. Please see Table 2 for the participants’ descriptions and Table 3 for details of comorbidities.

**Table 2:** Description of patients’ characteristics (n = 148).

Characteristic	Frequency (%)
Age in years mean (SD) 66.1 (17.5)	-
Settings	
Hospital 1	73 (49.3)
Hospital 2	75 (50.7)
Sex	
Male	67 (45.3)
Female	81 (54.7)
Type of ICU	
Medical	73 (49.3)
Surgical	43 (29.1)
Mixed	32 (21.6)
Diagnosis	
Sepsis	74 (50.0)
Septic shock	74 (50.0)
Having a urinary catheter (Foley’s catheter)?	
Yes	54 (36.5)
No	94 (63.5)

ICU = intensive care unit, SD = standard deviation.

**Table 3:** Charlson Comorbidity Index among patients with sepsis and septic shock (n = 148).

CCI item	Frequency (%)	
	Yes	No
Has the patient had a myocardial infarction?	26.4	73.6
Did the patient have congestive heart failure?	20.3	79.7
Did the patient have a peripheral vascular disease?	10.1	89.9
Did the patient have a cerebrovascular accident?	26.4	73.6
Did the patient have dementia?	5.4	94.6
Did the patient have a chronic obstructive pulmonary disease?	10.1	89.9
Did the patient have a connective tissue disease?	7.4	92.6
Has the patient had a peptic ulcer disease?	1.4	98.6
Did the patient have diabetes mellitus?	57.4	42.6
Did the patient have a moderate to severe chronic kidney disease?	32.4	67.6
Did the patient have hemiplegia?	20.3	79.7
Did the patient have leukaemia?	2.0	98.0
Did the patient have lymphoma?	0.7	99.3
Did the patient have a solid tumor?	11.5	88.5
Did the patient have liver disease?	7.4	92.6
Did the patient have acquired immunodeficiency syndrome (AIDS)?	0	100
Did the patient have hypertension?	68.2	31.8

Seventy-eight (52.7%) patients died within 30 days from diagnosis with sepsis and/or septic shock from both settings. Ninety-six percent of deaths occurred in the hospital (75 out of 78), and 3 patients died at home within 30 days of discharge. The results showed that the 30-day mortality rate of patients with sepsis was 26 (35.13%) and 52 (70.27%) for patients with septic shock. The chi-square test of independence showed a significant difference in 30-day mortality rate between patients with sepsis and patients with SS: [X2(1, N = 148) = 18.32, *P* < .001]. A phi coefficient was used to assess the strength of the relationship: Φ = .35, *P* < .001 (see Table 4).

**Table 4:** Mortality statistics of patients with sepsis and septic shock (n = 148).

Classification category	Thirty-day mortality status for		χ^2^ statistics	
	Dead, N = 78 (52.7%)	Alive, N = 70 (47.3%)	χ^2^	P
Type of ICU			3.0	.233
Medical ICUs	41 (56.2%)	32 (43.8%)		
Surgical ICUs	18 (41.9%)	25 (58.1%)		
Mix ICUs	19 (59.4%)	13 (40.6%)		
Diagnosis			18.3	<.001
Sepsis	26 (35.1%)	48 (64.9%)		
Septic shock	52 (70.3%)	22 (29.7%)		

ICU = intensive care unit, P = power (alpha level), χ^2^ = Chi-square test result.

Blood culture was performed for 118 (79.7%) patients, and urine culture was performed for 121 (81.8%) patients. Twenty-seven (22.9%) of positive blood cultures had gram-positive bacteria, and 16 (13.6%) cultures had gram-negative bacteria. The positive urine cultures had 28 (23.1%) gram-negative bacteria cultures. Coagulase-negative staphylococci were the most prevalent in the positive blood cultures (18, 15.3%), followed by 16 (13.6) samples positive for gram-negative bacteria. *Escherichia coli* was the most common in urine cultures (14.9%). Cultures were performed for all patients, but the type of culture was based on the patient’s source of suspected infection (see Table 5).

**Table 5:** Microbiology culture description of patients with sepsis and septic shock (n = 148).

Microbiology culture source	Frequency (%)								Coagulase-negative staphylococci
	Performed	Positive	Gram-positive	Gram-negative	Fungus	Mixed-bacterial growth	Mixed-bacterial growth including Candida species	Parasite	
Blood culture	118 (79.7)	46 (39.0)	9 (22.9)	16 (13.6)	3 (2.5)	-	-	-	18 (15.3)
Urine culture	121 (81.8)	47 (38.8)	2 (1.7)	28 (23.1)	13 (10.7)	4 (3.3)	-	-	-
Skin/soft tissue/wound culture	16 (10.8)	13 (81.3)	6 (37.5)	2 (12.5)	3 (18.7)	2 (12.5)	-	-	-
Sputum culture	56 (37.8)	33 (58.9)	1 (1.8)	20 (35.7)	8 (14.3)	2 (3.6)	2 (3.6)	-	-
Cerebrospinal fluid culture	8 (5.4)	2 (25.0)	2 (25.0)	-	-	-	-	-	-
Central venous catheter tip culture	1 (07)	1 (100)	-	1 (100)	-	-	-	-	-
Peritoneal fluid culture	1 (0.7)	0	-	-	-	-	-	-	-
Pleural fluid culture	7 (4.7)	2 (28.6)	1 (14.3)	-	-	-	1 (14.3)	-	-
Stool culture	8 (5.4)	2 (25.0)	-	1 (12.5)	-	-	-	1 (12.5)	-
Gallbladder Pile	1 (0.7)	0	-	-	-	-	-	-	-

Binary logistic regression was performed to assess for significant factors associated with mortality. Nine possible predictors were included in the model as follows: sepsis or septic shock, presence of a urinary catheter (Foley catheter), blood culture results as negative or positive, total SOFA score, history of having a solid tumor, mean arterial pressure at time of diagnosis, sex, age, and ICU type. All 148 patients were included in the analysis. A test of the full model including the 9 selected variables as predictors was statistically significant, X^2^(11) = 68.22, *P*-value < .001, indicating that LL (goodness of fit model) was significantly lower for the full model than for the null model. The strength of association between these variables was moderate: Nagelkerke’s R^2^ = .5, and Cox and Snell’s R^2^ = .4. The model correctly predicted patients’ 30-day mortality 78.4% of the time.

The Wald ratio for the variable of total SOFA score was significant, X^2^(df = 1) = 19.2, *P* < .001. This result showed that the odds of dying within 30 days of being diagnosed with sepsis and septic shock tend to be high for patients with higher total SOFA scores. The Wald ratio for the variable of history of having a solid tumor was significant, X^2^(df = 1) = 4.3, *P* = .04. This result showed that the odds of dying within 30 days of being diagnosed with sepsis and septic shock tend to be high for patients with solid tumors and metastatic tumors (Table 6).

**Table 6:** The results of binary logistic regression for 30-day mortality predictors among patients with sepsis and septic shock (n = 148).

Predictors	B	Wald Chi^2^ test	P	Exp (B)	95% CI for exp(B)	
					Lower	Upper
Total SOFA score	.41	19.25	<.001*	1.50	1.25	1.80
History of having a solid tumor	1.28	4.31	.04*	3.61	1.07	12.13
Age	.02	3.03	.082	1.02	.99	1.05
Gender	−.44	.98	.32	.64	.26	1.54
Mean arterial pressure	.02	1.76	.19	1.02	.98	1.06
Having sepsis or septic shock	.44	.26	.61	1.56	.28	8.64
Having a urinary catheter (Foley’s catheter)	.07	.03	.87	1.01	.44	2.61
The result of blood culture as negative or positive	.63	1.25	.26	1.88	.62	5.73
ICU type	−.40	.51	.47	.67	.22	2.00

B = regression coefficient, ICU = intensive care unit, P = power (alpha level), SOFA = Sequential Organ Failure Assessment.

* Significant value.

## 4. Discussion

A total of 148 patients with sepsis and/or septic shock were admitted to the selected ICUs during the data collection period. Seventy-eight (52.7%) of patients died within 30 days of being diagnosed with sepsis and septic shock. This mortality rate is comparable to the 28-day mortality of 50.5% among patients with sepsis and septic shock in Macedonia.^[7]^ However, it was higher than reported in a study conducted in Thailand, which was 21%.^[9]^ This is because their study focused on community-acquired sepsis among hospitalized patients. In future studies, it would be beneficial to differentiate between community-acquired sepsis and hospital-acquired sepsis.

The findings showed that 35.1% of the patients with sepsis alone died within 30 days of being diagnosed with sepsis, which was in line with previous study findings, which showed that the 30-day mortality of patients with sepsis was 37.58%.^[20]^ In accordance, another study revealed that the 30-day mortality rate of patients with severe sepsis was 27.5%.^[21]^ The 30-day mortality rate of the patients with sepsis reported in the present study was less than that found in a study conducted in a surgical ICU in Thailand, which was 55% for the overall 28-day mortality rate of adult patients with severe sepsis.^[22]^ In contrast, it was greater than the rate of 23% reported by Heldens et al^[8]^ for 28-day mortality. The difference in mortality rates could be attributed to variations in sepsis definition, comorbidities, types of infection, treatment protocol, and seasonal differences. Therefore, the period and season of data collection and patients’ clinical attributes need to be carefully considered when collecting data and when interpreting the results of individual studies.

The highest 30-day mortality rate was among patients who were admitted in mixed-type ICUs (59.4%), followed by medical ICUs (56.2%) and surgical ICUs (41.9%), with no significant statistical difference between them. However, the mix-type ICU in the current study was used to admit patients who had the most critical medical and surgical illnesses, which might lead to the highest mortality rate than the other types of ICUs. The present study enrolled patients from different types of ICUs, which enabled comparison between them.

The present study results showed that the significant predictors of 30-day mortality were solid tumors and total SOFA score. This was consistent with a previous study’s findings that the SOFA score could predict in-hospital mortality.^[23]^ This finding was congruent with a study conducted in the United Kingdom^[24]^ in which SOFA was the best predictive model for 30-day mortality. In general, having a higher SOFA score implies more failing organs or higher severity of an organ failure; this could explain the significant relationship between 30-day mortality and SOFA score. In addition, having a solid tumor can increase the mortality rate of patients with sepsis/septic shock. A similar finding was reported among 176 patients with cancer in a tertiary care center in Lebanon,^[25]^ in a large analysis study in the United States (n = 119,379),^[26]^ and in Brazil.^[27]^ In contrast, the mortality rate of patients with sepsis without cancer was higher than for patients with cancer in a retrospective cohort study in the United States of America.^[28]^ The researchers contributed this to the advances in sepsis treatment among patients with cancer.

The findings of the present study showed that the following variables were not significant predictors of the 30-day mortality: having sepsis or septic shock, having a urinary catheter (Foley’s catheter), and having negative or positive blood culture, history of having a solid tumor, mean arterial pressure, sex, and age. However, the findings above were supported by researchers who found that age was not a significant predictor of 28-day mortality among patients with sepsis and septic shock in 1 ICU in Turkey.^[29]^ Also, the result of the present study was supported by other researchers who indicated that increased age, hospital-acquired sepsis, and remaining hypotensive after receiving vasopressors^[30]^ were not significant predictors of 30-day mortality among patients with sepsis. In contrast, septic shock significantly predicted 30-day mortality of adult candidemia patients in an ICU of a tertiary care medical center in South Korea.^[31]^ However, their study was retrospective and included 126 patients with candidemia only.

The relatively small sample size of patients with sepsis and septic shock in the present study might affect the results of mortality prediction, given that 9 possible predictors were included in the logistic regression analysis. Patients with sepsis who have long hospital stays might develop other conditions that increase the risk of mortality other than sepsis alone.^[32]^ Seasonal variation of sepsis could affect the findings because this study’s data collection was performed between February and June. It seems sepsis is more prevalent in winter.^[7]^ This was also supported by a study that showed that sepsis could be affected by seasonal variation.^[33]^ However, the current study lays the background for further studies. The study design can be biased when considering patients with long hospital stays. It was reported that patients with sepsis who stayed for a long period inside hospitals might develop other conditions that increased the risk of mortality other than sepsis alone.^[32]^

It is important to teach them about the prognosis and outcomes of patients with sepsis and septic shock. Increasing awareness about the mortality of patients with sepsis and septic shock can help nurses and physicians to focus on improving the overall quality of health care of patients with sepsis and septic shock.^[34]^ The findings of the present study imply the need to undertake additional research studies to further understand sepsis and septic shock among adult patients in ICUs in the Jordanian health care context. Furthermore, qualitative studies need to be conducted to explore the impact of sepsis on patients and health care settings. It is recommended that sepsis screening in ICUs be improved using early warning systems and sepsis response teams.

It is recommended that ongoing staff development programs be undertaken, including teaching and training nurses and physicians with special knowledge and skills about assessing and treating adult patients with sepsis and septic shock. Moreover, health care policies and procedures concerning sepsis need to be stated and adopted in the hospitals of Jordan, with a focus on improving screening, treatment, and outcomes of patients with sepsis and septic shock.

## 5. Conclusions

This study demonstrated that the 30-day mortality rate for patients with sepsis and septic shock in 2 tertiary hospitals in Jordan was notably high, with 52.7% of patients succumbing to the condition. Mortality was significantly higher among patients with septic shock compared to those with sepsis alone, with a 70.27% mortality rate for septic shock patients. The study identified key predictors of mortality, including higher Sequential Organ Failure Assessment scores and a history of solid tumors. These findings underscore the importance of early diagnosis and aggressive treatment for sepsis, especially in high-risk patients with elevated SOFA scores or underlying cancer. The study’s results highlight the critical need for ongoing efforts to improve sepsis management in ICUs. Introducing sepsis response teams and using early warning systems could significantly reduce mortality by enabling earlier detection and intervention. Furthermore, the findings call for more in-depth research to differentiate the outcomes between community-acquired and hospital-acquired sepsis in order to better target treatment strategies.

## Acknowledgments

The authors extend their appreciation to Princess Nourah bint Abdulrahman University Researchers Supporting Project number (PNURSP2024R444), Princess Nourah bint Abdulrahman University, Riyadh, Saudi Arabia.

## Author contributions

**Conceptualization:** Saleh Al Omar, Jafar Alasad Alshraideh, Khalid Al-Mugheed.

**Formal analysis:** Jafar Alasad Alshraideh.

**Investigation:** Islam Oweidat, Mohammad Al Qadire.

**Methodology:** Mohammad Al Qadire, Atika Khalaf, Yasmeen Abu Sumaqa.

**Resources:** Atika Khalaf.

**Project administration:** Yasmeen Abu Sumaqa.

**Supervision:** Amany Anwar Saeed Alabdullah, Saleh Al Omar.

**Visualization:** Sally Mohammed Farghaly Abdelaliem.

**Writing – original draft:** Sally Mohammed Farghaly Abdelaliem.
